# Pancreatic Cystosis in Two Adolescents with Cystic Fibrosis

**DOI:** 10.1155/2016/5321785

**Published:** 2016-03-24

**Authors:** Elpis Hatziagorou, Asterios Kampouras, Maria Sidiropoulou, Andreas Markou, Athanasia Anastasiou, John Tsanakas

**Affiliations:** ^1^Paediatric Pulmonology and CF Unit, 3rd Paediatric Department, Hippokration Hospital, Aristotle University of Thessaloniki, 49 Konstantinoupoleos street, 54642 Thessaloniki, Greece; ^2^Magnetic Resonance Imaging Department, Hippokration Hospital, 54642 Thessaloniki, Greece

## Abstract

We present pancreatic cystosis in two adolescents with cystic fibrosis, a 13-year-old girl and an 18-year-old boy. In pancreatic cystosis, which is a rare manifestation of CF, the pancreatic parenchyma is replaced with multiple cysts of different sizes. Pancreatic cystosis is mainly an imaging based diagnosis and frequent follow-up should be recommended.

## 1. Introduction

Cystic fibrosis (CF) is an autosomic recessive inherited disorder affecting about 70.000 of people worldwide. Its main pathogenetic feature is a dysfunction of epithelial cells, which leads to the typical clinical triad: pancreatic exocrine insufficiency, chronic obstructive pulmonary disease, and elevated sweat chloride levels. Pancreatic exocrine insufficiency affects about 85% of patients with cystic fibrosis, being the most common gastrointestinal manifestation. Decreased production of pancreatic enzymes leads to fat malabsorption, with cystic fibrosis patients often presenting with malnutrition, steatorrhea, and fat soluble vitamin deficiency [1]. Cysts in the pancreas among CF patients are a rather common finding [2], whereas cysts larger than 1 cm are very rare [3]. In pancreatic cystosis, the pancreatic parenchyma is completely replaced by multiple cysts of different sizes, with abnormal pancreatic tissue [4]. We report two cases of pancreatic cystosis in two adolescents: a 13-year-old girl and an 18-year-old boy with cystic fibrosis. Over the past years significant progress has been made in the field of cystic fibrosis medications, with pancreatic enzymes, H2-histamine inhibitors in order to reduce gastric acidity and enhance fat absorption, and aggressive antimicrobial and anti-inflammatory treatment, along with CFTR potentiators starting to appear as potential drugs leading to a great improvement in life expectancy of CF patients. Despite the rapid progress in CF therapy, it is important for the clinicians to bear in mind that, even with such progress in the field of CF treatment, an extremely rare condition, such as pancreatic cystosis, can still be encountered and in this direction the evaluation and follow-up progress are highlighted.

## 2. Case 1

A 13-year-old girl with a past medical history of CF was admitted to our clinic for annual review.

She was diagnosed with CF at the age of 7 months, due to failure to thrive, with sweat test (Cl^−^: 114 mEq/L) and molecular testing (genotype: ΔF508del/ΔF508del).

The girl was not colonized with* Pseudomonas*; spirometry was within normal limits (FVC: 102% predicted and FEV_1_: 99% predicted). Her weight has been stable over the years in the 10th centile (41 kgr at the age of 13) with her height in the 25th (153 cm). She was on pancreatic enzymes and A, D, E, and K vitamins, due to pancreatic insufficiency.

**Figure 1:**
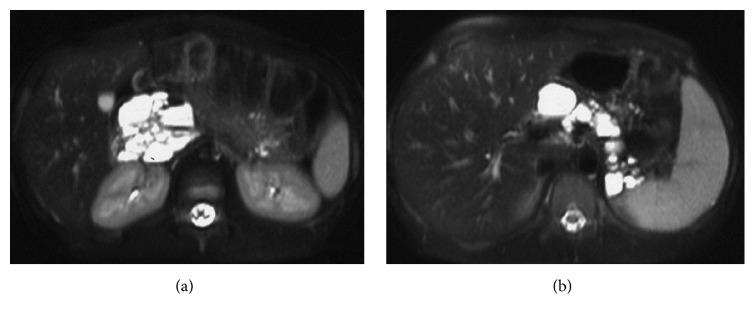
(a) T2-weighted fat-saturated transverse MR image obtained at the level of the pancreatic head shows multiple, high signal cysts of varying size in the expected region of the head of the pancreas without identifiable pancreatic tissue. (b) T2-weighted fat-saturated transverse MR image obtained at the level of the body and tail of the pancreas reveals complete replacement by multiple, bright-signal cystic masses of varying size with thin and smooth walls.

**Figure 2:**
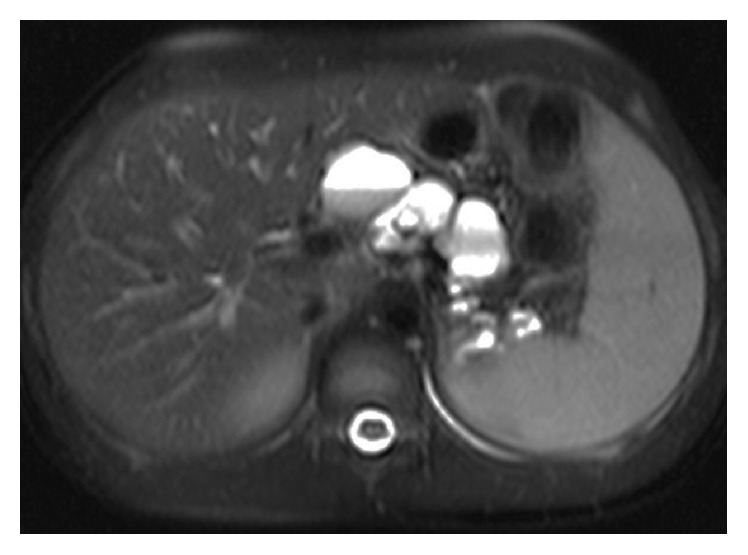
T2-weighted fat-saturated transverse MR image also shows multiple high signal, cystic lesions of various size at the expected region of the body of the pancreas. Note the fluid-fluid level in the largest cyst.

**Figure 3:**
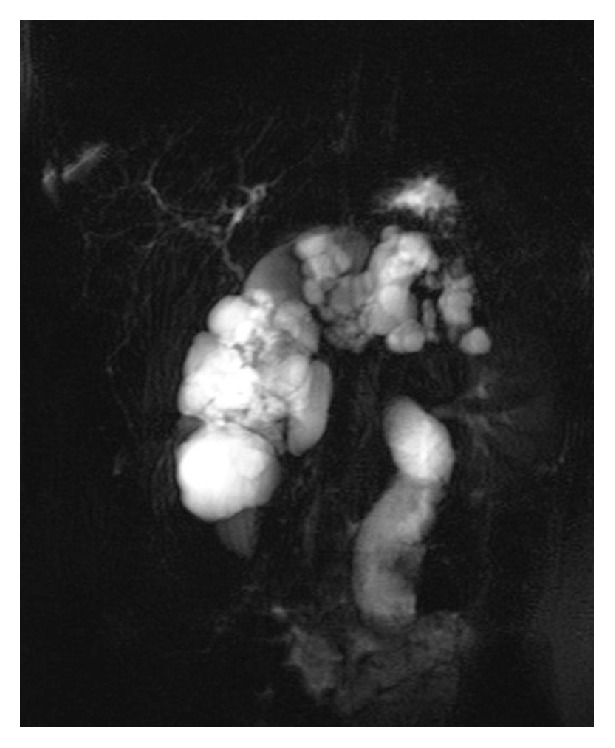
MRCP. T2-weighted MR image—HASTE (Half-Fourier Acquisition Single-Shot Turbo Spin Echo) sequence—demonstrates complete replacement of the pancreas by multiple cysts of variable size with thin and smooth walls. There is no communication of cysts with pancreatic ducts.

At the age of 11 years, during her annual review, abdominal ultrasound had revealed hyperechoic pancreas with multiple cysts along the peripancreatic duct with an average diameter of 3 cm per cyst. Further imaging with MRI confirmed the ultrasound findings. MRI findings revealed complete replacement of the pancreatic tissue by multiple cystic lesions of various sizes, with thin and smooth walls. The cystic lesions showed high signal intensity on T2-weighted images and low signal intensity on T1-weighted images and ranged in diameter (max 4.6 cm (Figure 1(a)). The largest cyst that appeared homogenous on the initial exam presented a fluid-fluid level on the first MRI follow-up two years later. Initial MRI findings revealed one cyst anterior to the right kidney, but MRI follow-up after two years showed that a new cyst anterior to the previously described cyst had been formed. In comparison, the diameter of a cyst at the tail of the pancreas had increased from 2.5 cm to 3.1 cm whereas the diameter of a cyst at the splenic hilum had decreased from 2.2 cm to 1.7 cm (Figure 1(b)). On the first follow-up MRI, some cysts demonstrated fluid-fluid levels due to hemorrhage (Figure 2). T2-weighted MR image—HASTE (Half-Fourier Acquisition Single-Shot Turbo Spin Echo) sequence—demonstrated complete replacement of the pancreas by multiple cysts of variable size with thin and smooth walls. No communication of cysts with pancreatic ducts was found (Figure 3). Oral Glucose Tolerance Test (OGTT) was normal.

## 3. Case 2

An 18-year-old boy with a history of cystic fibrosis was admitted to our clinic for his annual review.

The diagnosis was confirmed at the age of 3 months, with sweat test (Cl^−^: 128 mEq/L) and molecular testing (ΔF508/G542X).

The boy has been chronically colonized with* Pseudomonas aeruginosa* with moderate lung impairment (FVC: 80.8% predicted, FEV_1_: 68.0%, FEF50: 46.2% predicted, and LCI: 14.7). The boy also had pancreatic insufficiency, with fat soluble vitamins along with pancreatic enzymes being administered per os.

**Figure 4:**
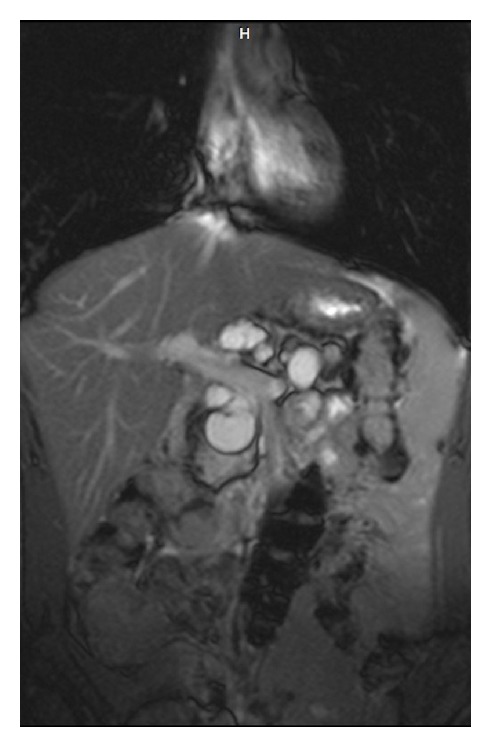
T2-weighted coronal MR image—TRUFI (True Fast Imaging with steady-state-free precession) sequence—obtained at the level of the portal-splenic venous confluence shows multiple high signal cysts of varying size replacing the pancreas.

One year ago, ultrasound revealed four cysts at the head and tail of his pancreas, whereas this years' ultrasound showed deterioration, with many cysts along the pancreatic duct. MRI findings confirmed the diagnosis of pancreatic cystosis, showing multiple bright-signal cysts of varying size (max 2.1 cm), which completely replaced pancreatic tissue. A cyst in the expected region of the neck of the pancreas appeared less bright on T2-weighted images than the other cystic lesions and showed high signal intensity on T1-weighted images due to protein content (Figure 4). All the other cysts in the expected region of the pancreas showed marked hypointensity on T1-weighted images. No cysts were seen in organs other than the pancreas. Oral Glucose Tolerance Test (OGTT) was normal.

## 4. Discussion

Cystic fibrosis is a common inherited multisystemic disorder affecting exocrine glands, with pancreatic insufficiency as a common manifestation, while pancreatitis is rarely seen in patients with CF. Nearly all patients with cystic fibrosis have pancreatic insufficiency and many of them present with failure to thrive, steatorrhea, and fat soluble vitamin deficiency.

Regarding pancreatic imaging, four different findings have been reported in cystic fibrosis patients despite their pancreatic function [5]. In the first pattern, pancreatic tissue is partly replaced by fibrofatty tissue. In the second one, pancreatic tissue is fully replaced by fibrofatty tissue. Atrophy of the pancreas is reported in the third pattern, whereas pancreatic cystosis is the fourth one. The first three findings are observed often in patients with cystic fibrosis (16%, 42%, and 24% of patients, resp.) [5], whereas pancreatic cystosis is highly uncommon.

The state in which pancreas is filled with macrocysts is called pancreatic cystosis [1]. Development of cysts has been related to bicarbonate transport [1]. Bicarbonate transport defect leads to low water in pancreatic secretions and high concentration of proteins, and pancreatic secretions ectasia of the pancreatic ducts along with inflammation. The increased fluid pressure proximal to the obstruction causes the inspissation of secretions which leads to the expansion of pancreatic ducts and their subsequent transformation into cysts of various sizes [1, 5, 6]. The fluid content of the cysts is mainly serous with amylase and protein levels being equivalent to the level of serum. So far, no cases of malignant transformation within pancreatic cystosis have been reported, but there are a few reports of mucinous adenomas in pancreatic cysts in CF patients [7].

Ultrasound and MRI findings include complete replacement of the pancreas by cysts of varying size, ranged from 0.5 to 1.2 cm in diameter, which may cause anterior displacement of the portal venous structures and the superior mesenteric artery [4]. MRI is the most accurate and helpful imaging method for evaluating the cysts and the other abdominal structures that may be compressed by large cysts. Cystic lesions can be assessed for size, signal intensity, nodules or solid foci, fluid-fluid level, and wall enhancement after gadolinium administration. The presence or absence of identifiable pancreatic tissue is also estimated.

There are several possible diagnoses for pancreatic cystic lesions and the differential diagnosis includes pancreatic pseudocysts, polycystic kidney disease, and von Hippel-Lindau disease with pancreatic involvement, microcystic adenoma, mucinous cystic neoplasm, and lymphangioma. On the basis of imaging findings and our patients' age and underlying disease, these differential diagnoses are safely ruled out and a diagnosis of pancreatic cystosis is made without necessity of biopsy [8, 9]. The number and location of the cysts and the absence of any signs of inflammation or previous trauma make the diagnosis of pseudocyst highly unlikely. Polycystic disease and von Hippel-Lindau disease also can be excluded on the basis of the normal appearance of the kidneys. Additionally, pancreatic involvement in polycystic disease differs pathologically from cystic fibrosis in that the main pancreatic ducts form multilocular cavities and the acini remain intact. Pancreatic microcystic adenoma in elderly women and mucinous cystadenoma (may be of high signal intensity on T1-weighted images due to the presence of mucin) is a disease seen in middle-aged women. Lymphangioma consists of cysts, which can show hemorrhage, but the involvement of the pancreas to this extent without disease in other abdominal organs is sufficient to exclude this diagnosis. Biopsy is considered unnecessary among patients with pancreatic cystosis. No standard treatment protocol exists for pancreatic cystosis [8]. Surgical approaches include drainage for large symptomatic cysts, by endoscopic or open gastro or duodenocystostomy, percutaneous drainage or aspiration, and partial or total pancreatectomy. In case of severe abdominal discomfort, total or partial surgical resection should be considered. If a patient reports symptoms related to an increase in volume of the pancreatic macrocysts (imaging based) and drainage or aspiration is performed, the quality of the fluid must be thoroughly examined for amylase, proteins, carcinoembryonic antigen (CEA) levels, and serum CA19-9 in order to exclude the possibility of a malignant mucin-producing neoplasm [4, 8, 10]. Although the overall risk of cancer in CF patients is similar to that of the general population, there is an increased risk of digestive tract cancer. For this reason, persistent or unexplained gastrointestinal symptoms deserve careful investigation and monitoring [11]. Besides, the diagnosis and treatment of malignant or potentially malignant pancreatic tumors will become challenging with the increase in life expectancy of patients with CF. It is therefore important to exclude features such as wall enhancement after gadolinium enhancement in order to rule out other coexistent cystic neoplasms. MR imaging offers precise anatomical depiction of pancreatic morphology and demonstrates subtle variations in the morphologic appearance of the pancreas in patients with CF for correlation with clinical course and progression of disease in cystic fibrosis.

In both of our patients the entire pancreas is replaced by many cysts. Both of our patients are asymptomatic; they have not complained of abdominal pain of discomfort. On MRI, the cystic lesions show low signed density when compared with the liver or the spleen on T1-weighted images and high signal intensity on T2-weighted images. There is no restricted diffusion. T1-hyperintense and T2-signal void cyst are suggestive of hemorrhagic content. Pancreatic resection also seemed contraindicated because the cysts surprisingly showed no symptoms. Besides, fecal fat during cyst progression was measured and did not deteriorate. Both of the patients did not seem to need more pancreatic enzymes daily; their *z*-scores for height and weight remained stable and endocrine function of their pancreas (Oral Glucose Tolerance Test) remained stable over the time course of the progression of the cysts, indicating no clinically significant deterioration in their pancreatic function. However, a six-month follow-up with ultrasound and if possible, yearly MRI are necessary. Ultrasound is useful and recommended for follow-up. However, MRI provides more diagnostic information about the characteristics of pancreatic cysts and is preferred in order to diagnose pancreatic cystosis. In a systematic review of the literature regarding the imaging of pancreatic cystic lesions [12], Jones et al. found that MRI had a sensitivity and specificity of 91.4–100.0% and 89.7%, respectively, in the assessment of main pancreatic duct communication. This observation can be very useful in cystic fibrosis patients that develop pancreatic cystosis in the context where ultrasonography does not provide subsequent data on the exact cyst position.

In conclusion, if pancreatic macrocysts are clearly found on imaging in a patient with cystic fibrosis, an imaging based diagnosis of pancreatic cystosis should be made and close follow-up should be recommended [3, 8, 9].
